# Unique presentation of congenital cataract concurrent with microcornea, microphthalmia plus posterior capsule defect in monozygotic twins caused by a novel *GJA8* mutation

**DOI:** 10.1038/s41433-018-0277-y

**Published:** 2018-11-29

**Authors:** Hongfang Zhang, Zhenji Chen, Kaiwen He, Pingjun Chang, Yinying Zhao, Xiufeng Huang, Jin Li, Zibing Jin, Yun-e Zhao

**Affiliations:** The Eye Hospital of Wenzhou Medical University, School of Ophthalmology and Optometry, The State Key Laboratory of Ophthalmology, Optometry and Vision Science, 325027 Wenzhou, China

**Keywords:** Mutation, Disease genetics

Congenital cataracts are the most common diseases which account for 10–30% of blindness in children [1]. Multiple genetic mutations contribute to the progression of this genetically heterogeneous and complex disease. Among the reported causative congenital cataract mutations, approximately one quarter are connexin genes, including Connexin 46 which is encoded by *GJA3* and Connexin 50 which is encoded by *GJA8* [2].

**Fig. 1:**
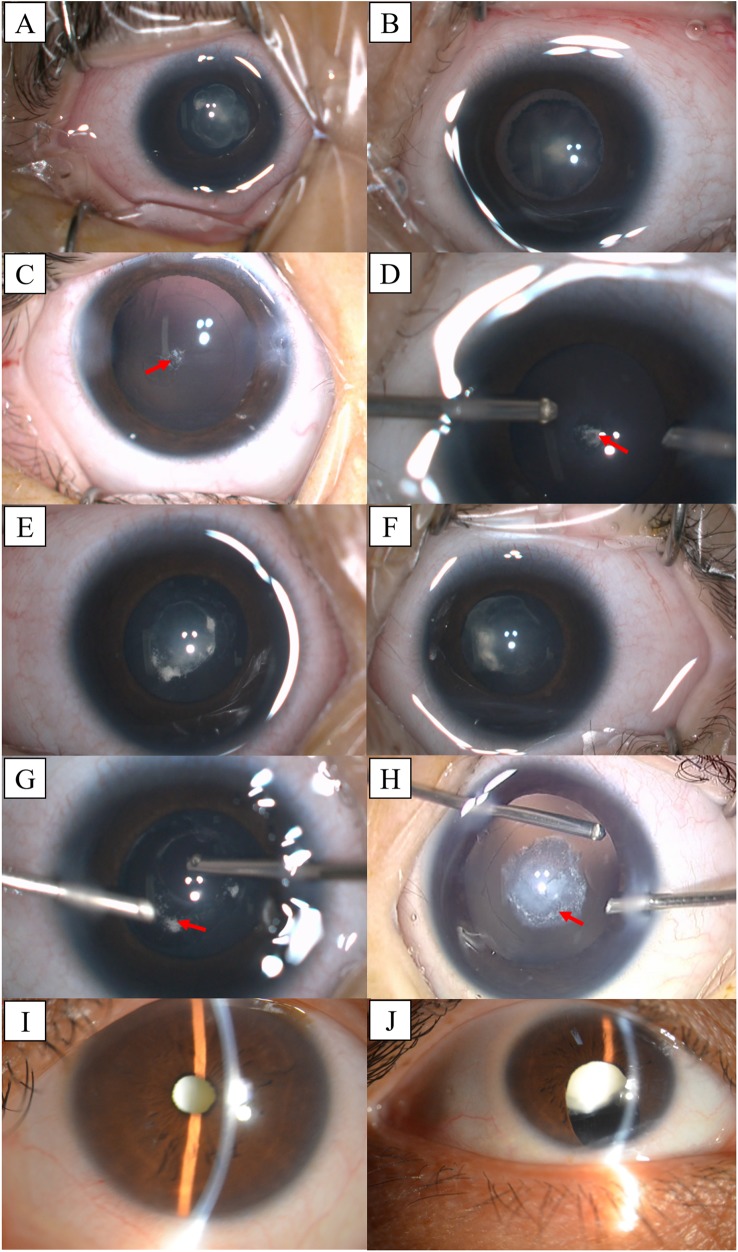
Anterior segment photographs of the affected individuals

In this study, we encountered four generations of a Chinese family with bilateral congenital cataracts at the Eye Hospital of Wenzhou Medical University. Among the four affected individuals in this family, two are twin sisters. The twins were diagnosed with bilateral congenital cataracts with microcornea, microphthalmia and posterior capsule defect (PCD). Photographs were obtained from a video of the surgery and confirmed the ophthalmologist’s diagnosis. Anterior segment photographs of proband (Fig. 1A, B) and her twin sister (Fig. 1E, F) show nuclear cataracts. Their posterior capsule photographs (Fig. 1C, D, G, H) demonstrate posterior polar cataracts with posterior capsule defects. Photographs of their grandmother (Fig. 1I, J) show full cataracts.

**Fig. 2:**
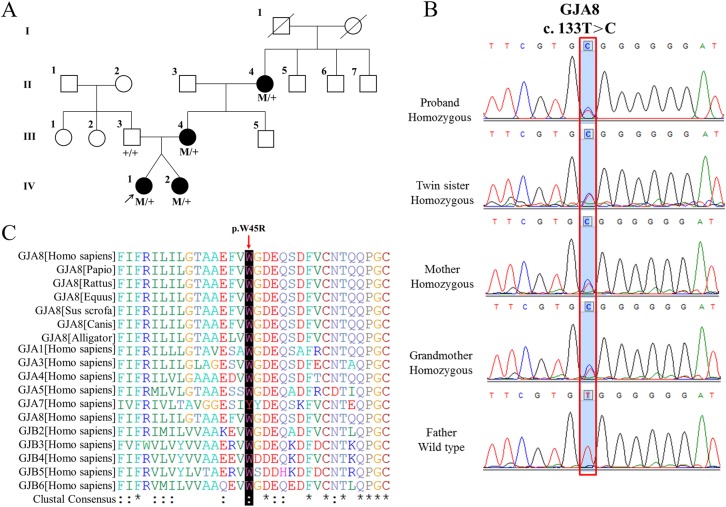
**A** Pedigree of four generations of the family with autosomal dominant cataracts. **B** Chromatograms showing the DNA sequence analysis in the *GJA8* mutation pedigree. **C** A multiple-sequence alignment of the amino-acid sequence in *GJA8* from various species and isoforms

To investigate the causative mutation in this family, we first performed whole exome sequencing on DNA from subject IV-1 (Fig. 2A) and identified a novel missense mutation, c. T133C, in *GJA8*. Sanger sequencing confirmed that the mutation co-segregated with all affected individuals and was not observed in the unaffected family member or in 100 unrelated controls (Fig. 2B). The mutation resulted in a missense amino-acid change, tryptophan to arginine, which was absent in dbSNP137, 1000 G, ESP6500 and ExAC databases. The arginine residue at position 45 is highly conserved across species and isoforms (Fig. 2C). Moreover, p.W45R is predicted to be pathogenic by SIFT (score 0.00 out of 1.00, “damaging”), Polyphen-2 (score 0.999 out of 1.000, “probably damaging”) and Mutation Taster (score 0.00 out of 1.00, “damaging”). Based on the above evidences, we can determine that the novel mutation (c.133 T > C, p.W45R) in *GJA8* is the pathogenic mutation in this family.

In conclusion, our study identifies a novel missense mutation in *GJA8* in a Chinese family with congenital cataracts using WES, thereby expanding the existing spectrum of *GJA8* mutations.
